# Selective Transarterial Embolization for Intractable Hematuria Due to Bladder Carcinomas: A Single-Center Experience

**DOI:** 10.5152/tud.2023.22224

**Published:** 2023-09-01

**Authors:** Azad Hekimoglu, Onur Ergun, Baki Hekimoglu

**Affiliations:** Department of Radiology, Diskapi Yildirim Beyazit Training and Research Hospital, Ankara, Turkey

**Keywords:** Bladder carcinoma, hematuria, embolization

## Abstract

**Objective:**

Malignant bladder neoplasms can cause life-threatening persistent hematuria. Selective transarterial embolization is an effective way to achieve hemostasis in such patients. The purpose of our study is to share our experience in these patients and to evaluate the short- and long-term effectiveness of this procedure.

**Methods:**

Twelve male patients with intractable hematuria due to bladder carcinoma were included in the study. A total of 17 selective transarterial embolization procedures (bilateral in 5 patients) were performed in 12 patients with microspherical particles and microcoils.

**Results:**

Complete control of bleeding was achieved in 9 patients after the procedures whereas the need for transfusion continued in 3 patients. Approximately 75% bleeding control was achieved during our average 6-month (4- to 12-month) follow-up period. After the procedure, the patients had mild complaints that lasted for a few days, such as pain (66%), fever (42%), and nausea (50%). No major complications occurred.

**Conclusion:**

Selective transarterial internal iliac artery embolization is a reliable method that can be used for the palliative treatment of intractable hematuria due to bladder carcinomas.

**Main Points**

- Bladder malignant neoplasms can cause life-threatening persistent hematuria.
- Transarterial embolization is a widely used minimally invasive method.
- Transarterial internal iliac artery embolization is a safe method used for the treatment of intractable hematuria in patients with bladder neoplasia.

## Introduction

Bladder malignant neoplasms usually cause persistent hematuria, which is an important life-threatening problem. In patients with severe bleeding, symptomatic anemia develops and there is a need for continuous transfusion. There are some forms of treatment available, such as medical management of acute bleeding, bladder irrigation with saline, alum or formalin, or bladder hydrodistension. However, these methods are not always effective and any surgical procedure may be associated with significant morbidity.^1-3^

Selective transarterial embolization of the internal iliac artery represents an alternative, safe, and effective way of achieving hemostasis in these patients and has been successfully applied for many years to treat bladder bleeding associated with terminal pelvic malignancy.^4^ Our aim in this study is to convey our experience in patients with persistent hematuria due to malignant bladder neoplasm who underwent selective transarterial embolization and to evaluate the applicability and short- and long-term effectiveness of this procedure in hemorrhagic urological emergencies.

## Material and Methods

Approximately 18 patients who underwent selective transarterial embolization treatment in the interventional radiology unit of our hospital with unstoppable hematuria due to bladder tumor between December 2018 and May 2021 were included in the study. Five patients were excluded from the study because their follow-up and treatment did not continue in our hospital and the data could not be reached. In addition, 1 of our patients was excluded from the study because the procedure could not be performed due to excessive tortuosity of bilateral iliac arteries. All 12 patients included in the study were male and the mean age was 72.6 years. Both right and left internal iliac arteries were embolized in 5 of our patients, and a total of 17 embolization procedures were performed. The pre- and post-procedure examinations of the patients, as well as the general condition of the patients after the procedure, were followed up from the patient files. Images of the patients were analyzed retrospectively from the image archive of hospital (Table 1).

This study has been approved by the Ethics Committee of the Diskapi Training and Research Hospital (protocol number: 67/01-2019). All methods were performed in accordance with the relevant guidelines and regulations and informed consent from all the patients has been taken.

### Transarterial Selective Embolization Procedure

**Figure 1. Digital Subtraction Angiography (DSA):**
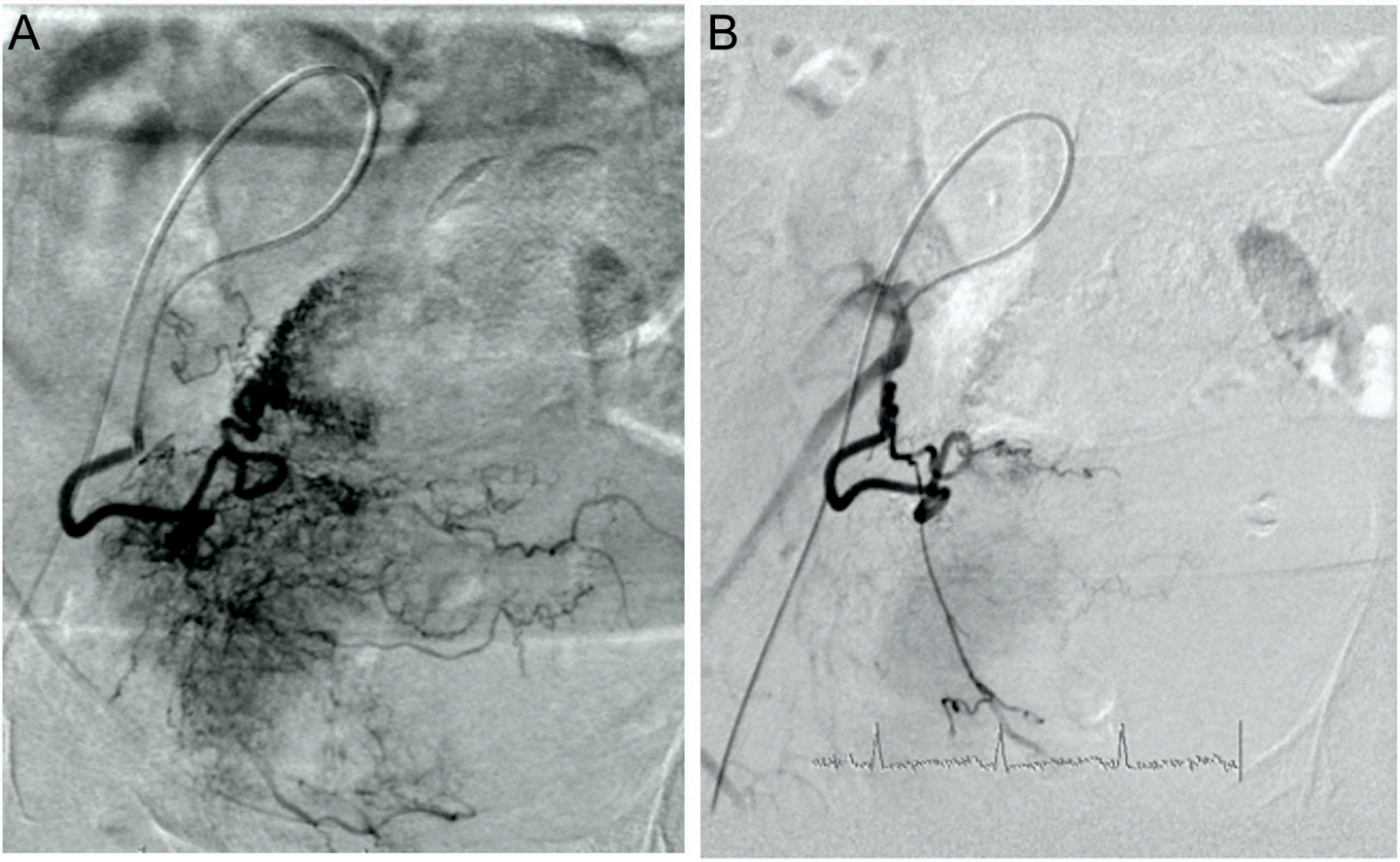
image of bladder cancer before (A) and after (B) embolization with selective catheterization.

After inserting the vascular sheath by entering through the main femoral artery, separate images were taken from the pelvic aorta with the help of a pigtail catheter, including both iliac arteries and their branches, and then selectively catheterizing the right and left internal iliac arteries. After determining the side with more mass staining, the anterior branch or branches of the internal iliac artery, which mainly provides mass vascularization, were identified by advancing with the help of a catheter and afterward superselectively catheterized with microcatheters. With the help of a microcatheter, the branch or branches that provide mass vascularization were embolized with microparticles (polyvinyl alcohol). The most commonly used microparticle size was 300-500 μm. In addition, 100-300 μm in 3 patients, 500-700 μm in 2 patients, and 700-900 μm in 1 patient were used according to vessel diameter and mass size. The process was terminated by embolizing the feeder branch from the proximal by means of multiple microcoils due to the significant decrease in mass staining in the images taken (Figure 1).

## Results

Unilateral embolization was performed in 7 patients, including left internal iliac artery branches in 5 patients and right internal iliac artery branches in 2 patients. In the images taken, embolization to bilateral internal iliac artery branches was performed in 5 patients because the bladder mass was large and it was fed from internal iliac artery branches on both sides. Contralateral internal iliac artery embolization was performed after 4 days in 1 patient, after 3 days in 3 patients, and after 24 days in 1 patient. Complete control of bleeding was achieved in 9 patients after the procedure. The patients had an average daily blood requirement of 4 (2-9) transfusion units before the procedure. However, optimal bleeding control could not be achieved in 3 patients (1 month after embolization and 2 months after embolization) and the need for transfusion continued. In our mean follow-up period of 6 months (4-12 months), approximately 75% of bleeding control was achieved.

In addition, a significant difference was observed between the mean hemoglobin (7.55) and hematocrit (23.05) levels before the procedure, and the hemoglobin (9.77) and hematocrit (29.52) levels were measured approximately 1 month after the procedure (*P *< .05). Of course, the hemoglobin and hematocrit levels measured before the procedure may not reflect the actual levels since they are under transfusion.

Although there was no significant complication after the procedure, 8 patients had short-term pain in the groin that lasted for 2-3 days (66%), 5 patients had fever (42%), and 6 patients had nausea (50%) that lasted for a few days. One of our patients died about 4 months after the procedure for reasons independent of the procedure.

## Discussion

It is known that bladder irrigation, Helmstein balloon compression, and cystoscopic clot evacuation are among the most protective methods used in persistent hematuria due to bladder malignant neoplasia.^5^ Although surgical interventions such as internal artery ligation or salvage cystectomy are the last resort for control of persistent hematuria, these procedures are associated with high morbidity and mortality.^6-8^ Internal iliac artery embolization was first performed in 1974 and is widely used in the treatment of persistent hematuria due to malignant neoplasia of the bladder.^3,4,6,8,9^ There have been few reports of serious complications of this procedure, such as necrosis of the bladder after internal iliac artery embolization in patients with severe pelvic trauma. However, similar complications were not observed after hematuria treatment.^10,11^ Post-embolization syndrome (nausea, vomiting, gluteal pain, and fever due to tissue necrosis), gluteal pain, bacterial sepsis, gait disturbances, Brown–Sequard syndrome, and skin necrosis are also rare complications of transarterial embolization.^12,13^ The most common complication, gluteal pain, is due to embolic material passing into the superior gluteal artery in the posterior part of the internal iliac artery. This complication of embolization has been largely prevented by selective catheterization of the anterior part of the internal iliac artery.^14^ In the study conducted by Pisco et al in 108 patients with uncontrollable bleeding due to pelvic neoplasms (bladder in 50, uterus in 39, ovary in 16, and prostate in 3), bleeding recurred in 33 patients, postembolization syndrome in 70 patients, and in 3 patients, there was transient acute tubular necrosis caused by contrast agent.^4^ More recently, in the study of Nabi et al,^15^ 50% of their patients had minor complications such as nausea, vomiting, and fever for an average of 2 days, with no other major complications. In our study, no serious complications were observed except for mild complications such as fever, pain, and nausea. One of our patients died 4 months after embolization, but it was due to causes other than embolization.

During the transarterial embolization procedure, the vascular structures that supply the mass are selectively embolized with microembolizing particles, and then these vascular structures are completely closed with microcoils. The purpose of not only closing the vascular structures with coils but also embolizing them with microparticles is to prevent re-blowing of the vascular structures with collateral structures. In such a case, it is not possible to reach these vascular structures again. Some studies suggest bilateral embolization of the anterior branches of the internal iliac arteries, regardless of whether the bleeding point will be detected on angiography, in order to prevent the recurrence of collateral bleeding.^8,16,17^ In our study, bleeding control was achieved in our patients who underwent bilateral embolization. Bleeding recurred in 3 of 7 patients who underwent unilateral embolization. Two of the 3 patients with recurrent bleeding were embolized on the right and 1 on the left. Recurrence of bleeding is observed in both patients who underwent right embolization.

There are many published series showing that 70%-95% of bleeding control is achieved immediately after transarterial embolization.^2,4,5,9,14^ In the study conducted by Giovanni et al,^6^ it was stated that bleeding control was achieved in 81%. In another study, it is said that 100% success was achieved in bleeding control.^8^ In our study, no decrease was observed in the hemoglobin and hematocrit values of the patients after embolization in a short time, and 75% hematuria control was achieved, similar to the results obtained in studies performed in long-term follow-up.^6^ However, hematuria recurred in 3 (25%) patients after a short time and a transfusion requirement arose. Re-development of hematuria in these patients cannot be explained by collateral development in a short time. This may be due to possibly rapid re-neovascularization of the mass.

The patient population with malignant bladder neoplasia generally consists of the elderly and the mean age of our patients was 72.6. Vascular tortuosity increases in elderly patients,^18,19^ and selective vascular angiography becomes more difficult for embolization. As a matter of fact, the procedure could not be performed in one of our patients due to vascular tortuosity. In addition, it is common for microparticles to leak back and reflux to other vascular structures due to vascular structure disorder during embolization in these patients.

Some studies emphasize that embolization should always be done bilaterally if possible.^20,21^ Performing bilateral embolization for patients who do not need it brings additional risk to the patient as well as an additional cost. In our study, 5 patients (42%) required bilateral embolization. Unilateral embolization was successful in 58% of patients.

Among the limitations of the study are the retrospective nature of the study and the small number of patients with unstoppable hematuria due to malignant bladder neoplasia.

In conclusion, transarterial internal iliac artery embolization, which is used for the treatment of unstoppable hematuria in patients with malignant bladder neoplasia, is a safer and less invasive method, although sometimes minimal complications are seen.

## Figures and Tables

**Table 1.** Demographic and Clinical Features

Patient (procedure)	12 (17)
Age	72.6
Left iliac embolization	5
Right iliac embolization	2
Bilateral embolization	5
Fever	5 (42%)
Pain	8 (66%)
Nausea	6 (50%)
Hematuria (relapse)	3 (25%)
Hemoglobin g/dL (before)	7.55
Hemoglobin g/dL (after)	9.77
Hematocrit % (before)	23.05
Hematocrit % (after)	29.52
